# NMR metabolic fingerprints of murine melanocyte and melanoma cell lines: application to biomarker discovery

**DOI:** 10.1038/srep42324

**Published:** 2017-02-15

**Authors:** Arquimedes Paixão de Santana-Filho, Thiago Jacomasso, Daniel Suss Riter, Andersson Barison, Marcello Iacomini, Sheila Maria Brochado Winnischofer, Guilherme Lanzi Sassaki

**Affiliations:** 1 Departamento de Bioquímica e Biologia Molecular, Universidade Federal do Paraná, Cx.P 19046, CEP 81531-990, Curitiba, PR, Brazil; 2 Departamento de Química, Universidade Federal do Paraná, Cx.P. 19081, CEP 81531-990, Curitiba, PR, Brazil

## Abstract

Melanoma is the most aggressive type of skin cancer and efforts to improve the diagnosis of this neoplasia are largely based on the use of cell lines. Metabolomics is currently undergoing great advancements towards its use to screening for disease biomarkers. Although NMR metabolomics includes both 1D and 2D methodologies, there is a lack of data in the literature regarding heteronuclear 2D NMR assignments of the metabolome from eukaryotic cell lines. The present study applied NMR-based metabolomics strategies to characterize aqueous and lipid extracts from murine melanocytes and melanoma cell lines with distinct tumorigenic potential, successfully obtaining fingerprints of the metabolites from the extracts of the cell lines by means of 2D NMR HSQC correlation maps. Relative amounts of the identified metabolites were compared between the 4 cell lines. Multivariate analysis of ^1^H NMR data was able not only to differentiate the melanocyte cell line from the tumorigenic ones but also distinguish among the 3 tumorigenic cell lines. We also investigated the effects of mitogenic agents, and found that they can markedly influence the metabolome of the melanocyte cell line, resembling the pattern of most proliferative cell lines.

Melanoma presents a major challenge in medical practice, due to difficulty in diagnosis, poor response to available therapies and high incidence in western populations^1^. The study of melanoma has greatly benefited from the use of cell lines, which allow the investigation of progressive changes in the metabolism that correlate with the tumorigenic process^2^. As a non-destructive technique, Nuclear Magnetic Resonance (NMR) can be used in the preliminary characterization of both water-soluble and lipid metabolites; multivariate analysis methodologies such as Principal Component Analysis (PCA), can subsequently point out the main contributors that distinguish cell lines or experimental data sets^3^. Though not as sensitive than Mass Spectrometry (MS)-based methods, NMR-based metabolomics is currently being broadly applied to investigate metabolic profiles, due to both the ongoing development of inverse detection and cryogenic methods that greatly increases sensitivity, and the use of 2D and selective 1D methodologies which permit unequivocal identification of the metabolic profile by overcoming problems involving overlap. Due to the high variation in metabolic phenotype in humans^4^, cell line based studies are a basic requirement for pointing out the main metabolic changes linked to tumorigenic development. Established cell lines provide a model in which the biological variability is small enough to allow the development and application of metabolomic methodologies that, allied to proper experimental design, provide reliable results, generating highly reproducible metabolic fingerprints which lead to a molecular classification of the disease stage or subtype^5^. One of the most used cell line models for melanoma is the murine B16-F10^6^,^7^, which represents a highly metastatic stage of melanoma, originally obtained from a tumour spontaneously generated in C57BL/6 J mice which was then subjected to serial *in vitro* cultivation and *in vivo* passages in the same organism. Another melanoma model includes the Tm1 and Tm5 melanoma cell lines, which were established by submitting the melanocyte cell line Melan-a to sequential cycles of substrate-adhesion impediment^8^, of the two, Tm5 having a higher tumorigenic potential when injected *in vivo* and a lower doubling time *in vitro*^8^. Although established melanocytes cell lines are important for performing comparative studies^9^, mitogenic agents such as tumour-promoting phorbol-ester must be added to the growth media in order to stimulate melanocyte growth^10^,^11^. Their mechanism of action involves the activation of a family of proteins named protein kinase C (PKC), which in turn play important roles in mediating cell growth, differentiation, and tumour promotion^12^. Regarding these effects, some studies relate irregularities in melanocyte morphology to their being cultured in the presence of phorbol esters^13^. The current study used NMR, Multivariate analysis and RT-qPCR techniques to characterize both water-soluble and lipid extracts from the following set of murine cell lines: non-tumorigenic melanocytes (Melan-a), Tm1 and Tm5 melanomas and B16-F10, a highly metastatic melanoma cell line. The aims of this study are to apply 2D multiplicity-edited HSQC NMR techniques in order to obtain fingerprints from lipid and aqueous extracts from the four cell lines and identify and determine the relative amounts of altered metabolites, using ^1^H NMR spectroscopy allied to multivariate analysis. We also investigated the effect of mitogenic agents on the metabolic profile of the melanocyte cell line.

## Results

### ^1^H and multiplicity-edited ^1^H-^13^C HSQC NMR Spectroscopy

**Figure 1:**
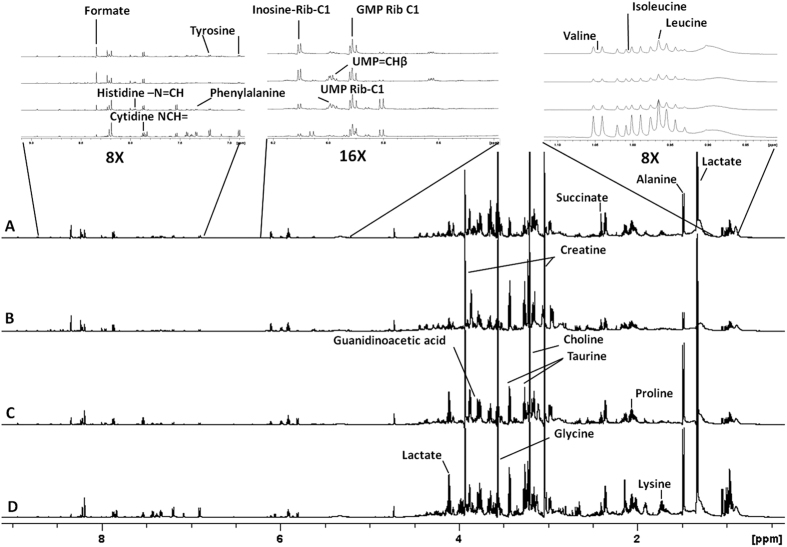
^1^H NMR spectra of aqueous extracts obtained from mouse cell lines. Melan-a (**A**), Tm1 (**B**), Tm5 (**C**) and B16-F10 (**D**). The chemical shifts are relative to the internal standard TSP (δ = 0.00 ppm). For more detailed assignments, see Supplementary Table S1.

**Figure 2:**
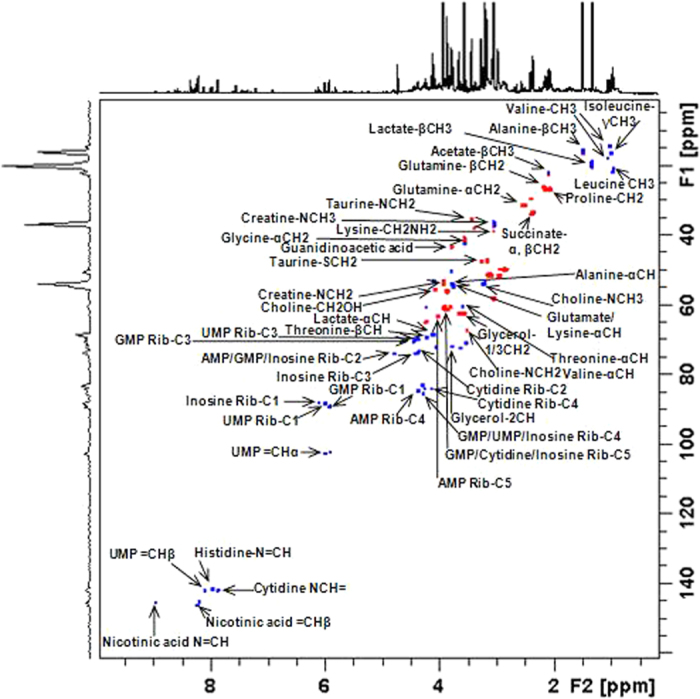
Partial 2D multiplicity-edited HSQC NMR correlation map of aqueous extract obtained from the Tm5 cell line with the principal assignments. The positive phase (blue) corresponds to CH and CH_3_ correlations, and the negative phase (red) corresponds to CH_2_ correlations.

**Figure 3:**
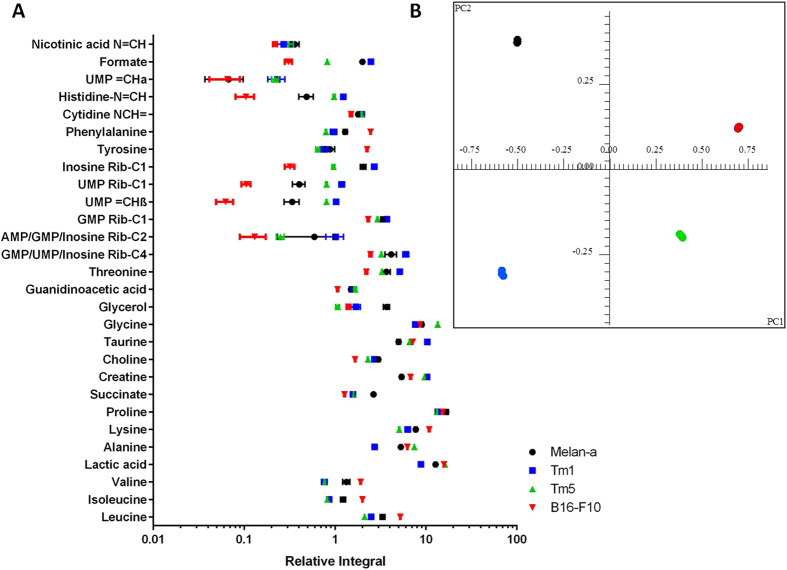
(**A**) Relative concentrations of aqueous metabolites in the extracts from the 4 cell lines namely Melan-a (black), Tm1 (blue), Tm5 (green) and B16-F10 (red). The relative intensities were obtained from the integration of the ^1^H spectra and normalized to TSP peak area integral; **(B)** Scores plot (PC1 vs PC2) of the PCA analysis performed.

The ^1^H NMR spectra of the aqueous extracts obtained from the four cell lines showed many overlapping signals (Fig. 1 and Supplementary Table S1), and thus 2D experiments (TOCSY, multiplicity-edited HSQC and HMBC) were performed. The resonance assignments, performed based on both 1D and 2D NMR experiments, with the aid of literature data^14^,^15^,^16^,^17^ and the Human Metabolome Database^18^,^19^, allowed the identification of 64 metabolites assignments(Fig. 2), 28 being in a range that allowed the assessment of the relative averaged concentration (Fig. 3A).

It is evident upon simple inspection that all the metabolites demonstrated differences in concentration among the cell lines, and that differences in some metabolites concentrations were linked to major alterations in metabolism which occur in tumorigenic cells, examples of which are higher concentrations of lactic acid and lower concentrations of glycerol seen in the metastatic cell lines (except for the Tm1 cell line)^20^. However, for some metabolites, the increase or decrease in concentration was not always linked to the tumorigenic potential of the respective cell line^21^.

### Multivariate and gene expression analysis

**Figure 4:**
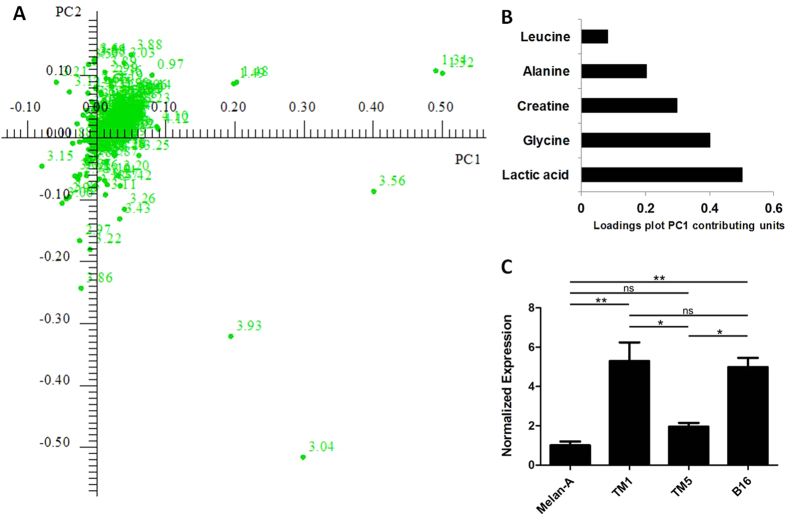
(**A**) 2D Loadings plot of the PCA analysis; (**B**) Loadings plot contributing units of main metabolites for PC1; (**C**)RT-qPCR quantification of mRNA coding for GAMT. *P < 0.05; **P < 0.01; ns = statistically not different.

In order to determine how well these metabolites correlate with the tumorigenic potential of the cell lines, a principal component analysis was carried out on the ^1^H NMR spectra obtained from the cell extracts. The 4 cell lines were separated into 4 distinct groups on the scores plot (Fig. 3B), and both the first (PC1, horizontal axis) and second (PC2, vertical axis) principal components contributed to the differentiation, resulting in a cumulative explained variance of 72.36% and 89.33%, respectively. As expected, the melanocyte cell line Melan-a was completely separated from the both Tm5 and B16-F10 cell lines on PC1. In addition to distinguishing the smaller differences exhibited by the Tm1 cell line in PC1, PC2 was able to further distinguish these cell lines from each other. The 1D loadings plot of the PCA analysis (see Supplementary Fig. S1) pointed out the main buckets, and thus, the corresponding peaks on the spectra that contributed significantly to the separation of each principal component. The higher the intensity of the peaks in its pseudo-1D spectrum, the greater the contribution of the peak in each PC, thus enabling us to point out the main metabolites in the aqueous extract that contributed to the separation of the four cell lines in both PC1 and PC2. An analysis of both the 1D loadings plot (Supplementary Fig. S1) and the 2D loadings plot, demonstrated that the buckets which exerted the most influence on differentiation in both PC1 and PC2 were attributed to peaks assigned to the metabolite creatine. In order to measure the influence of these peaks on separation, the absolute values of 2D loadings plot (Fig. 4A) were obtained for both PC1 and PC2, and plotted as shown in Fig. 4B and Supplementary Fig. S2. These data together pointed toward an imbalance in creatine metabolism. To confirm this hypothesis, gene expression experiments were carried out. RT-qPCR assays proved that mRNA expression levels encoding the enzyme GAMT (Guanidinoacetate-Methyl-Transferase), which is responsible for catalyzing the final step of creatine synthesis, were highly altered among the 4 cell lines, the lines Tm1 and B16-F10 expressed at least 2.5 times more mRNA when compared to Tm5 and ~5 times when compared to Melan-a (Fig. 4C). All 3 tumorigenic cell lines showed mRNA levels at least 2 times higher than those of the melanocyte cell line.

**Figure 5:**
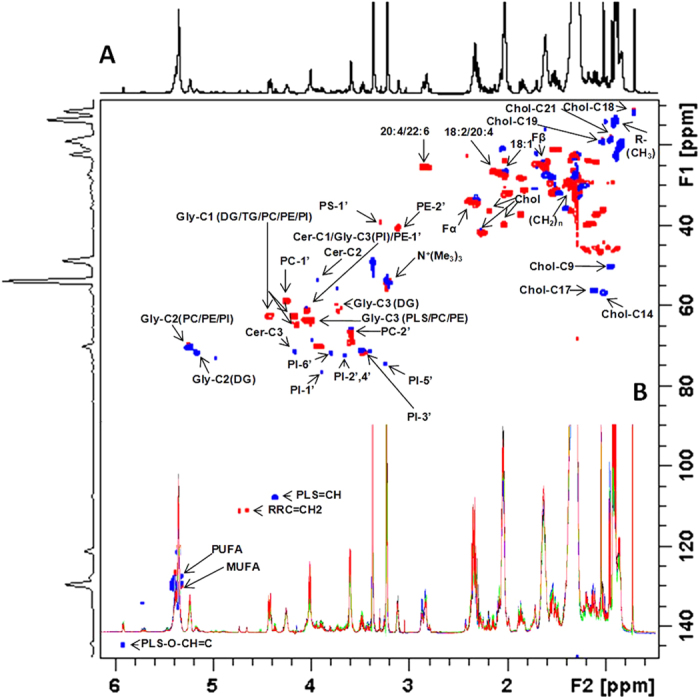
(**A**) Partial 2D multiplicity-edited HSQC NMR correlation map of the lipid extract obtained from the Tm5 cell line with the principal lipid assignments. The positive phase (blue) corresponds to CH and CH_3_ correlations, and the negative phase (red) corresponds to CH_2_ correlations; **(B)** Overlaid ^1^H NMR spectra of the 4 cell lines: Melan-a (black), Tm1 (blue), Tm5 (green) and B16-F10 (red). The chemical shifts are relative to the internal standard TMS (δ = 0.00 ppm). For more detailed assignments, see Supplementary Table S2.

Regarding the lipid extracts, the ^1^H NMR spectra proved to be much less overlapped than that of the aqueous extract (Fig. 5A and Supplementary Table S2); in addition 2D experiments allowed us to identify the majority of the lipid classes present in the extracts from the 4 cell lines (Fig. 5B), with the aid of literature data^22^,^23^,^24^. In contrast to the aqueous metabolites, no significant differences were found. Even when we applied PCA analysis to the spectra, it was impossible to distinguish the tumorigenic cell lines from the non-tumorigenic one (Supplementary Fig. S3).

### Analysis of the effect of PMA on the metabolomic profile of melanocytes

**Figure 6:**
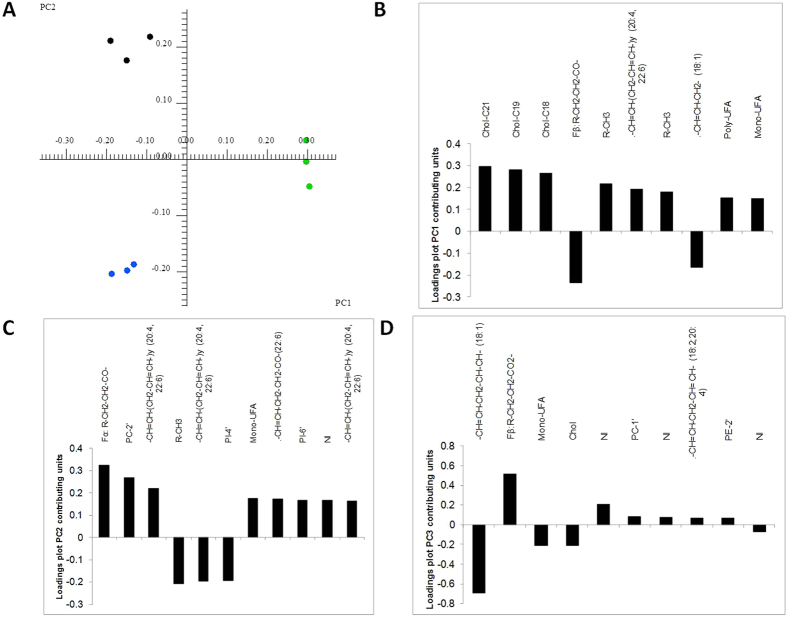
(**A**) Scores plot (PC1 vs PC2) of the PCA analysis performed with the ^1^H NMR spectra of lipid extracts of mouse cell line Melan-a (under the three different growth conditions, namely MA (black), MA-24 (blue) and MA-72 (green); **(B)** Loadings plot contributing units of main metabolites for PC1; **(C)** Loadings plot contributing units of main metabolites for PC2; (**D**) Loadings plot contributing units of main metabolites for PC3. NI – Not identified.

As previously stated, some studies associate the use of phorbol esters, such as PMA, with an altered differentiation of melanocyte cells^13^, by means of a mechanism that involves the activation of PKC^25^. Consequently, we aimed to verify whether these morphological changes could be linked to the metabolism of structural lipids and thus influence the discrimination pattern obtained from the ^1^H NMR analysis of the lipid extracts. In order to do this, the Melan-a cell line was cultivated under the following three conditions; 1) with PMA present in the media during the entire time (MA), 2) with the cell line cultivated in a medium without PMA for 24 hours (MA-24), and 3) where the cell line was cultivated for 72 h without the presence of PMA in the medium (MA-72). ¹H NMR and principal component analyses were then carried out on the lipid extracts of the cells. The first principal component explained 45% of the variance, the second 72%, and the third 84%, cumulatively. Even the explained variance in the first PC not being ideal, the 3 conditions were well discriminated on the scores plot of both PC1 vs PC2 (Fig. 6A) and PC2 vs PC3 (Supplementary Fig. S4), and the loadings plot of PC1, PC2 and PC3 pointed out the main contributors among lipid classes, thus indicating the main lipid classes that can be influenced by the use of phorbol esters (Fig. 6B–D). In the first PC, the main NMR peaks contributing to separation were assigned to cholesterol, the Fβ from fatty acids, C20:4 or C22:6 and C18:1. In the second PC, Fα from fatty acids, phosphatidylcholine and C20:4 and C22:6 fatty acids were the main contributors, and in the third PC C18:1 fatty acids and Fβ were the main signals contributing to distinguish the cell lines. Since the higher separation was achieved in PC1, we were able to determine that the effects of PMA with reference to the lipid composition of the cells were mainly, but not uniquely, related to the cholesterol content. We also performed ^1^H NMR analysis on the aqueous extracts obtained from MA-24 and MA-72 treatments (Supplementary Fig. S5), and observed that the main metabolites with differences in concentration between the treatments were choline and lactic acid, with the levels of choline being higher in MA-72 as compared to MA, and the opposite occurring in the lactic acid levels (Supplementary Fig. S6). Lysine, proline, creatine and threonine also showed significant concentration differences among the 3 growth conditions.

The multiplicity-edited HSQC experiment provided very useful information intended for analyzing the glycerol moiety in the phosphoglycerolipids, via phase editing multiplicity assignments of its C1, C2 and C3 residual glycerol carbons, phospholipid-specific polar head group carbons, and chemical shifts of specific fatty acid carbons. Also, the ^1^H-^31^P HMBC allowed the identification of phospholipids presents in the extracts.

## Discussion

Several studies in the literature employ ^1^H NMR in order to obtain metabolomic profiles of extracts from both tissues and cell lines^16^,^26^,^27^,^28^, and the application of these methods combined with multivariate analysis to characterize the metabolome of several neoplasias is a continuously developing field in cancer research^29^,^30^,^31^,^32^,^33^. The majority of these studies, however, provide only 1D ^1^H or homonuclear 2D NMR assignments, which can result in erroneous assignments due to the high signal overlap in ^1^H NMR spectra obtained from aqueous extracts. Thus, the 2D NMR HSQC fingerprints presented in the current study constitute a reliable tool for resolving ambiguities arising from signal overlap in 1D ^1^H NMR spectra. To the author’s knowledge, this is the first time that 2D ^1^H-^13^C multiplicity-edited HSQC NMR correlation maps of both aqueous-soluble and lipid-soluble metabolites were obtained from tumorigenic cell lines.

Metabolic characterization of the malignant transformation process can reveal molecules associated with tumorigenic development^34^,^35^,^36^,^37^. For the current work, NMR analysis was performed on both aqueous and lipid-soluble extracts of the following cell line types: a non-tumorigenic melanocyte cell line, two tumorigenic melanoma cell lines, established from the first, and a highly metastatic melanoma cell line. The results obtained made it possible to both identify at least 28 aqueous-soluble metabolites and approximately 20 lipid classes and measure their relative concentrations. PCA analysis performed on the ^1^H spectra allowed the identification of the main metabolites that contributed to differentiation, and the 2D multiplicity-edited HSQC NMR fingerprints proved to be very useful for unambiguously identifying the main NMR peaks. The main metabolites in the aqueous extracts which showed differences in concentration related to the progressive increase in the tumorigenic potential of the cell lines were lactate, glycine, creatine, alanine and leucine; among these, creatine influenced separation on of both PC1 and PC2 principal components, as is evident from an analysis of the loadings plot. Thus, RT-qPCR experiments were carried out which showed that the mRNA coding for GAMT was highly altered among the four cell lines, demonstrating at least double the values when the melanocyte cell line was compared to the tumorigenic ones. Although the larger creatine/creatinine ratios in the Tm1, Tm5 and B16-F10 cell line could be caused by any of several factors, the most likely is that the concentration of these metabolites is related to the well-known elevated metabolite rate in malignant cells, playing an essential role in energy storage^38^. Creatine kinase and citrate synthase demonstrate increased activity when cells are depleted of tissue creatine^39^. Creatine is implicated in adenosine triphosphate synthesis due to its participation in the phosphocreatine energy system. Creatine and phosphocreatine are also involved in the shuttling of adenosine triphosphate from the inner mitochondrial membrane into the cytosol, acting as a spatial energy buffer^40^. The higher mRNA expression levels of GAMT in the three tumorigenic cell lines in comparison to GAMT levels observed in Melan-a cells confirmed alterations in the synthetic biochemical pathway of this metabolite in melanoma cells.

The use of mitogenic agents is widespread in studies involving cell lines, (whether of human and murine origin), and the Melan-a cell line is no exception. Stranzl *et al*.^41^ evaluated the effects of PMA on both hormone responsive (ER+) and hormone unresponsive (ER-) mammary tumour cell lines. Their results showed that when PMA was added to the culture medium, the ER+ cells showed a significant increase in Low Density Lipoprotein Receptor (LDL-R) mRNA, suggesting that PKC was influencing delivery of exogenous cholesterol to cancer cells, through regulation of LDL-R mRNA levels. We have now found via multivariate analysis that the lipidome from Melan-a cell line showed alterations when PMA was added to culture media, and its effect was shown to be time-dependent. The main signals that contribute for differentiation were assigned to saturated and unsaturated fatty acids and cholesterol. Therefore, we can now hypothesize a similar role, in which PMA can mimic DAG and thus activate PKC^12^, which can in turn influence cholesterol levels. Analysis of the Melan-a cell line in the presence and absence of PMA demonstrated that the use of this tumour promoter can markedly influence lipid metabolism, causing it to resemble more closely the profile of highly proliferative cell lines. ^1^H NMR analysis of the aqueous-soluble extracts also corroborates this hypothesis, since the relative concentrations of lactic acid were higher in the Melan-a cell line which had been grown with PMA in the culture media the entire time. The use of PMA or other analogous mitogenic agents certainly influences cell metabolism and some cautions and considerations should be taken in this experimental condition.

In summary, the present study successfully applied NMR techniques in order to identify and determine the relative amounts of aqueous and lipid-soluble metabolites of both melanocyte and melanoma murine cell lines. Through PCA analysis it was possible to identify the main contributors which differentiate the 4 cell lines. The results showed that the metabolite which most contributes to differentiation in the aqueous extract obtained from the 4 cell lines on both PC1 and PC2 was creatine, as confirmed by gene expression assays. Therefore, we can state that there is an imbalance in creatine metabolism (Supplementary Fig. S7), such as that this metabolite pathway is more active when the tumorigenic potential increases between the cell lines.

Furthermore, this study demonstrated that the metabolomic profile of the melanocyte cell line is influenced by the use of mitogenic agents, such as PMA. Although not so evident as the concentration changes observed for aqueous metabolites, lipid metabolism changes between the Melan-a cell line grown with and without PMA added on culture media could be revealed by the use of multivariate analysis techniques, showing that the main alterations were related to the metabolic pathway of cholesterol. The 2D multiplicity-edited HSQC NMR fingerprints proved to be a useful tool for identifying and measuring different expression patterns of metabolites in both water soluble and lipid extracts, enabling us to identify potential progression biomarkers and thus contribute to the development of methods for the prognosis of melanoma.

## Methods

### Cell line and cell culture

Mouse melanoma cell lines (B16-F10, Tm1 and Tm5) and the immortalized murine melanocyte cell line Melan-a were kindly provided by Dr. Roger Chammas (Laboratory of Experimental Oncology, School of Medicine, University of São Paulo, SP, Brazil). Melan-a, Tm1 and Tm5 cells were grown in 75 cm^2^ tissue-culture flasks and maintained in RPMI medium supplemented with 5% (v/v) fetal bovine serum (FBS), both from Gibco (Pascagoula, MS, U.S.A.), 200 nM 12-*O*-tetradecanoyl phorbol-13-acetate - PMA (Sigma, St.Louis. MO), 100 U/mL penicillin and 100 μg/mL streptomycin (Gibco) in a humidified atmosphere containing 5% CO_2_ at 37 °C. Melanoma cell line B16-F10 was cultured under the same conditions, without PMA. The cells were grown until 75–80% density and then collected using 0.1% trypsin in PBSA containing 1 mM EDTA and centrifuged at 4000 rpm for 20 minutes (4 °C). The cell pellet was washed thrice with phosphate buffer, the supernatant discarded and the pellet subjected to the extraction process. We standardized a quantity of 15 mg for each cell line, which corresponded to approximately 4 × 75 cm^2^ tissue-culture flasks.

### Metabolite extractions

The cell pellets were subjected to lysis, being frozen with liquid nitrogen and returned to a 37 °C water bath several times. After being freeze-dried overnight, 15 mg of each cell line, was weighted and transferred to a glass tube with screw cap and Teflon (volume of 4 mL). The pellet was extracted using acetonitrile:water 1:1 (1 mL), vortexed for 1 minute and then kept for 10 min at room temperature (this procedure was repeated thrice), and then centrifuged at 10000 rpm for 6 min. The supernatant was collected and lyophilized. The pellet from the previous aqueous extraction procedure was then subjected to lipid extraction using CHCl_3_:MeOH 3:1 (v/v), being vortexed for 1 minute and then kept for 30 min at room temperature. After this, it was centrifuged at 10000 rpm for 20 min, the supernatant collected and the solvent dried under a stream of nitrogen^3^.

### NMR spectroscopy

MeOD, CDCl_3_, Deuterium oxide (D_2_O, 99.9% D) and 3-trimethylsilyl-^2^H_4_-propionic acid sodium salt (TSP) were purchased from Cambridge Isotope Laboratories, Inc. (Miami, U.S.A.) and from Sigma-Aldrich (St. Louis, MO). The samples obtained from the aqueous extraction were deuterium-exchanged by repeated dissolution in D_2_O, and the samples obtained from the lipid extraction were deuterium exchanged by repeated dissolution in MeOD-D_2_O (2:1). Both were freeze-dried overnight. Their spectra were obtained from solutions in D_2_O (580 μL plus 20 μL of TSP 1 mg/mL) or CDCl_3-_MeOD (3:1, 600 μL) at 30 °C, using TMS (Tetramethylsilane) or TSP as a reference (δ = 0.00 ppm). Spectra were obtained on a Bruker 400 MHz AVANCE III NMR spectrometer with a 5 mm BBI inverse probe and a Bruker 600 MHz ASCEND equipped with a QXI probe (Bruker Biospin, Germany). 1D ^1^H-NMR was carried out using enough scans to give a Signal/Noise (S/N) ratio of at least 2000/1 (90° pulse, relaxation delay = 4.0 s, number of time domain points = 65536, spectral width = 10.6541 ppm and acquisition time = 7.7 s). Experiments were performed without tube rotation and with the TMS or TSP signal at a medium width ranging from 0.8–1.0 Hz. Water suppression was obtained by presaturation, using the 1D zgpr pulse sequence, applying a low power continued wave irradiation before the first 90° pulse on channel 1, using a power level for presaturation of 44.98 -dBW (3.17 × 10^−5^ Watts). For multivariate analysis and estimation of metabolites concentration, the spectra were acquired using the noesygppr1d.2 pulse sequence, with 128 transients and a delay of 10.0 s (2.0 s on the analysis of lipid samples). 2D NMR experiments were carried out using multiplicity-edited ^1^H-^13^C HSQC, heteronuclear correlation via double inept transfer with decoupling during acquisition, using trim pulses in inept transfer with multiplicity editing during the selection step (hsqcedetgpsp.3), TOCSY, total homonuclear correlation via Hartman-Hahn transfer using MLEV17 sequence for mixing with a mixing time of 0.06 s (mlevphpr.2), and ^1^H-^13^C/^1^H-^31^P HMBC, heteronuclear correlation via zero and double quantum coherence optimized on long range couplings (hmbcgplpndqf and hmbcgpndqf) pulse sequences. The 2D experiments were recorded for quadrature detection in the indirect dimension, HSQC spectra were acquired using 128 scans per series of 2 K × 400 W data points with zero filling in F1 (4 K) prior to Fourier transformation^42^.

### Quantitative gene expression assays

GAMT (Guanidine-Acetate Methyl Transferase - EC 2.1.1.2) mRNA expression levels were quantified in murine cell lines Melan-a and B16-F10. For this, cDNA were synthesized using the ImProm II Reverse Transcription Kit (Promega), from 1 μg of total RNA, and using Oligo d(T)_18_ primers, according to manufacturer’s instructions. RT-qPCR reactions were performed in a StepOne Plus (Applied Biosystems, Foster city, CA, U.S.A.) thermal cycler, using SYBR Green PCR Master Mix (Applied Biosystems) and specific primers for the genes of interest. The equipment’s default cycling program was used (15 s at 95 °C and 1 min at 60 °C for 40 cycles). Amplification efficiency and data normalization were performed as previously described^43^, using ACTB as reference gene. A dissociation cycle was performed after each run to check for non-specific amplification or contamination. Primer sequences used were: GAMT sense: 5′-ATGTTTGAGGAGACGCAGG-3′; Antisense: 5′-AGGCATAGTAGCGGCAGT-3′; ACTB Sense: 5′-AAGATCAAGATCATTGCTCCTCC-3′; Antisense: 5′-CGTACTCCTGCTTGCTGATC-3′. All experiments were performed thrice, and the results were submitted to one-way ANOVA with Tukey in order to test for statistical significance. Proposed alterations on the biological pathway were drawn with the software PathVisio^44^.

### Analysis of the effect of PMA on the lipid profile of melanocytes

Melan-a cell line were grown in RPMI medium containing 200 nM of PMA during four passages. The medium was then removed and replaced with a medium which did not contain PMA. The cells were left for a further 24 and 72 hours of growth.

### Multivariate data analysis

Multivariate analyses were performed on the spectra obtained from both aqueous and lipid extractions. Spectra obtained from 3 to 5 independent experiments, each one with 3 or more technical replicates were assembled to build the bucket tables. For spectra obtained from aqueous extractions, the spectral width considered was from 0.50 to 10.00 ppm. After NMR spectral phase and baseline correction, each spectrum was data-reduced to 1183 regions of equal width (0.005 ppm) using the AMIX (Analysis of Mixtures) software package, version 3.8 (Bruker Biospin, Rheinstetten, Germany). The confidence level was 95.00%. For spectra obtained from lipid extractions, the spectral width considered was from 0.50 to 6.00 ppm. Each spectrum was data-reduced to 453 regions of equal width (0.005 ppm). The spectral region close to CHD_2_OD resonances (*δ* 3.33–3.38) was removed from all data sets prior to normalization and multivariate data analysis in order to remove deviations due to water suppression efficiency or solvent multiplicity inhomogeneities. Following a preliminary PCA analysis, the region corresponding to (CH_2_)_n_ (*δ* 1.20–1.40) and N^+^ (CH_3_)_3_ (*δ* 3.18–3.25) resonances was also removed from all datasets, mainly because minor peak shape deformations in this region interfered with PCA analysis, due to the high intensity of signals. All remaining frequency regions of the spectra were analyzed without scaling, in order to preserve natural differences in intensities from the datasets^3^. In order to evaluate the relative amounts of metabolites in the aqueous extracts, each FID was first multiplied by an exponential function with 0.60 Hz Lorentz line broadening prior to Fourier transformation. Spectra were then manually phased and baseline corrected and subsequently referenced using the chemical shift of TSP at 0.000 ppm. The integrals of the metabolites were then normalized by the integral of the TSP, and subsequently expressed as relative proportion of each metabolite. Data processing and integration were performed using the Software TOPSPIN version 3.1 (Bruker Biospin, Rheinstetten, Germany). Statistical significance of the differences between cell lines or treatments was calculated using 2-way ANOVA, followed by Tukey test. The p-values obtained were adjusted to account for multiple comparisons. Calculations were performed using Graphpad Prism 6.01.

## Additional Information

**How to cite this article**: Santana-Filho, A. P. d. *et al*. NMR metabolic fingerprints of murine melanocyte and melanoma cell lines: application to biomarker discovery. *Sci. Rep.* **7**, 42324; doi: 10.1038/srep42324 (2017).

**Publisher's note:** Springer Nature remains neutral with regard to jurisdictional claims in published maps and institutional affiliations.

## Supplementary Material

Supplementary Information
