# Supplementary material for: NMR metabolic fingerprints of murine melanocyte and melanoma cell lines: application to biomarker discovery
Source: Sci Rep. 2017 Feb 15;7:42324. doi: 10.1038/srep42324 (PMC5309734; doi:10.1038/srep42324)
Supplement: Supplementary Information [file srep42324-s1.doc]

Supplementary Information

**NMR metabolic fingerprints of murine melanocyte and melanoma cell lines: application to biomarker discovery.**

Arquimedes Paixão de Santana-Filho

Thiago Jacomasso

Daniel Suss Riter

Andersson Barison

Marcello Iacomini

Sheila Maria Brochado Winnischofer

Guilherme Lanzi Sassaki

Supplementary Figure S1

**Figure S1.** 1D Loading plot of PC1 (Black) and PC2 (Blue).

Supplementary Figure S2

**Figure S2.** Loadings plot contributing units of main metabolites for PC2.

Supplementary Figure S3

**Figure S3.** Scores plot (PC1 vs PC2) of PCA analysis performed with the ^1^H NMR spectra of lipid extracts of the cell lines: Melan-a (black), TM-1 (blue), TM-5 (green) and B16-F10 (red).

Supplementary Figure S4

**Figure S4.** Scores plot (PC2 vs PC3) of PCA analysis performed with the ^1^H NMR spectra of lipid extracts of the Melan-a cell line: MA (black), MA-24 (blue) and MA-72 (green).

Supplementary Figure S5


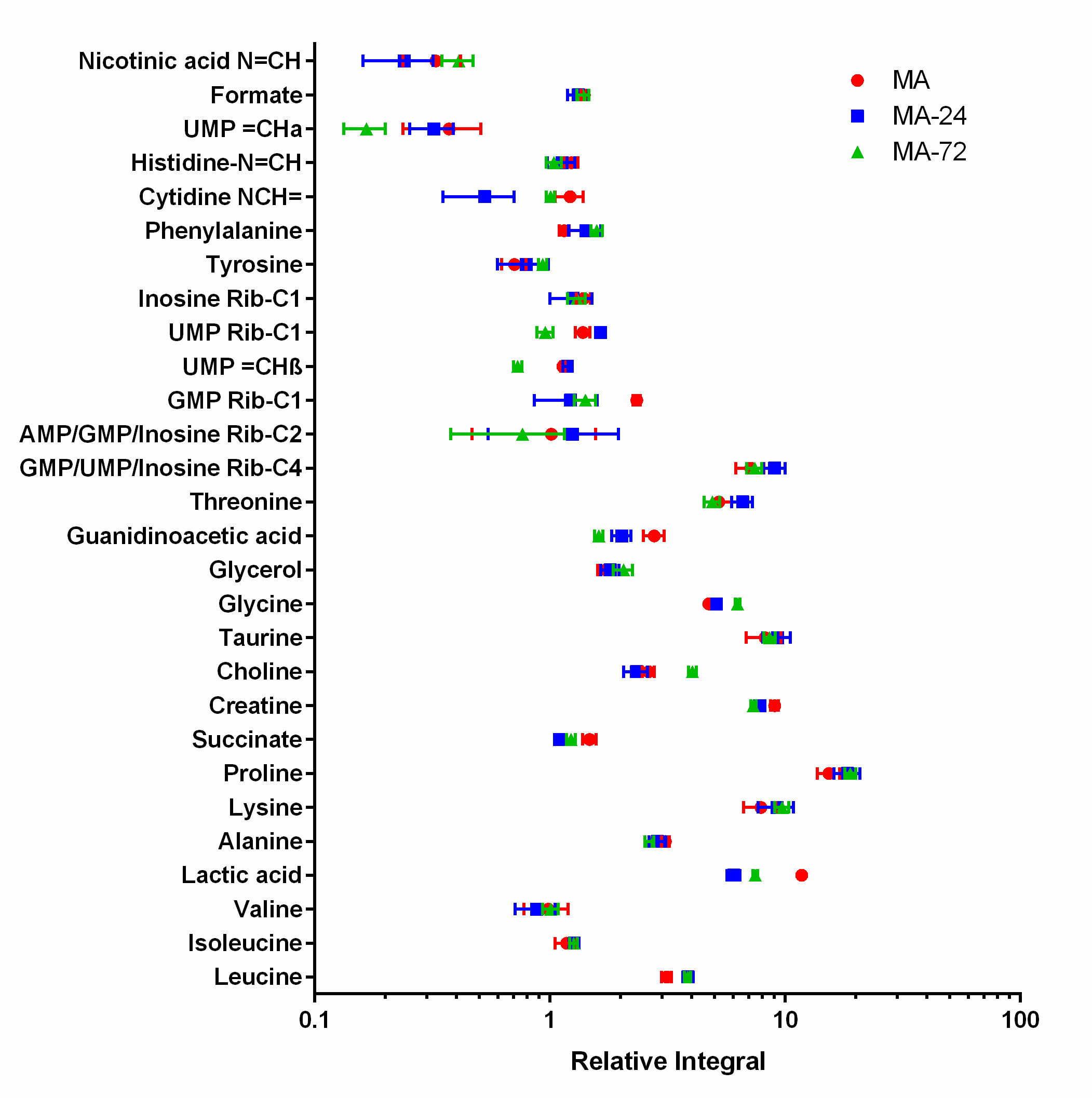


**Figure S5.** Relative concentrations of aqueous metabolites in the extracts from Melan-a cell line under the three different growth conditions, namely MA (red), MA-24 (blue) and MA-72 (green).

Supplementary Figure S6

**Figure S6**. Statistical significance of measured levels of aqueous metabolites in the extracts from Melan-a cell line under the three different growth conditions, and in the extracts obtained from mouse cell lines. Melan-a, Tm1, Tm5 and B16-F10.

Supplementary Figure S7

**Figure S7**. Schematic model of the main metabolites altered between the cell lines and treatments on both aqueous and lipid soluble extracts.

Supplementary Table S1

| Compound | ^1^H Chemical shift | ^13^C Chemical shift | 2D phase assignment |
| --- | --- | --- | --- |
| TSP | 0.00 (s) | 0.00 | CH_3_ |
| Isoleucine | 0.943 (t) | 11.29 | CH_3_ |
| Leucine | **0.966** (t) | 21.30 | CH_3_ |
| Leucine | 0.969 (t) | 22.25 | CH_3_ |
| Isoleucine | **0.996** (d) | 16.92 | CH_3_ |
| Valine | 1.014 (d) | 14.91 | CH_3_ |
| Valine | **1.046** (d) | 18.22 | CH_3_ |
| Lactic acid | **1.330** (d) | 20.37 | CH_3_ |
| Alanine | **1.4838** (d) | 16.42 | CH_3_ |
| Lysine | **1.718** (m) | 26.76 | CH_2_ |
| Proline | **2.063** (m) | 27.22 | CH_2_ |
| Acetate | 2.088 (m) | 22.45 | CH_3_ |
| Proline | 2.131 (m) | 27.30 | CH_2_ |
| Glutamine | 2.168 (m) | 26.53 | CH_2_ |
| Proline | 2.3597 (dt) | 33.90 | CH_2_ |
| Succinate | **2.406** (s) | 34.55 | CH_2_ |
| Glutamine | 2.525 (m) | 31.88 | CH_2_ |
| Creatine | **3.041** (s) | 37.24 | CH_3_ |
| Choline | **3.205** (s) | 54.19 | CH_3_ |
| TMAO | 3.223 (s) | 60.60 | CH_3_ |
| Betaine | 3.229 (s) | 54.38 | CH_3_ |
| Taurine | **3.261** (t) | 47.83 | CH_2_ |
| Taurine | 3.429 (t) | 35.68 | CH_2_ |
| Choline | 3.521 (m) | 67.78 | CH_2_ |
| myo-inositol | 3.535 (m) | 71.47 | CH |
| Glycine | **3.560** (s) | 41.76 | CH_2_ |
| Glycerol | **3.573** (m) | 62.85 | CH_2_ |
| Threonine | 3.585 (s) | 60.81 | CH |
| myo-inositol | 3.628 (m) | 72.78 | CH |
| Glycerol | 3.655 (dd) | 62.91 | CH_2_ |
| Glutamate/Lysine | 3.758 (m) | 55.04 | CH |
| Alanine | 3.786 (q) | 54.53 | CH |
| Glycerol | 3.787 (m) | 72.38 | CH |
| Guanidinoacetic acid | **3.792** (m) | 43.77 | CH_2_ |
| GMP/Cytidine/Inosine -Rib C5 | 3.876 (m) | 61.54 | CH_2_ |
| Creatine | 3.930 (s) | 54.23 | CH_2_ |
| AMP-Rib C5 | 4.028 (m) | 63.85 | CH_2_ |
| Choline | 4.063 (m) | 55.94 | CH_2_ |
| Lactate | 4.109 (q) | 68.84 | CH |
| Cytidine-Rib C4 | 4.136 (m) | 84.56 | CH |
| Threonine | **4.228** (m) | 69.79 | CH |
| GMP/UMP/Inosine Rib-C4 | **4.279** (m) | 85.89 | CH |
| Cytidine Rib-C2 | 4.353 (m) | 73.89 | CH |
| UMP Rib-C3 | 4.359 (m) | 70.35 | CH |
| AMP Rib-C4 | 4.368 (m) | 85.09 | CH |
| Inosine Rib-C3 | 4.424 (m) | 74.29 | CH |
| GMP Rib-C3 | 4.440 (m) | 70.76 | CH |
| AMP/GMP/Inosine Rib-C2 | **4.775** (m) | 74.38 | CH |
| GMP Rib-C1 | **5.912** (t) | 89.69 | CH |
| UMP =CHβ | **5.983** (m) | 103.02 | CH |
| UMP Rib-C1 | **5.997**(m) | 88.71 | CH |
| Inosine Rib-C1 | **6.105** (d) | 88.59 | CH |
| Tyrosine | **6.904** (d) | ND | CH |
| Tyrosine | 7.200 (d) | ND | CH |
| Phenylalanine | **7.335** (m) | ND | CH |
| Phenylalanine | 7.382 (m) | ND | CH |
| Phenylalanine | 7.428 (m) | ND | CH |
| Nicotinic acid | 7.599 (m) | ND | CH |
| Cytidine NCH= | **7.868** (d ) | 142.32 | CH |
| Histidine-N=CH | **7.959** (m ) | 141.98 | CH |
| UMP =CHα | **8.110** (d ) | 142.43 | CH |
| Nicotinic acid =CHβ | 8.231 (d ) | 146.62 | CH |
| Formate | **8.343** (s) | ND | CH |
| Nicotinic acid N-CH | 8.718 (m) | ND | CH |
| Nicotinic acid N=CH | **8.942** (m) | 145.83 | CH |

**Table S1**. ^1^H NMR assignments performed in the aqueous extracts of the cell lines. The chemical shifts are relative to the internal standard TSP (δ = 0.000 ppm). The bold ^1^H assignments represents the ones used in order to measure the relative amounts of each metabolite. TSP, 3-trimethylsilyl-^2^H_4_-propionic acid sodium salt; TMAO, trimethylamine N-oxide; GMP, Guanosine 5’-monophosphate; Rib, Ribose; UMP, Uridine 5’-monophosphate; Adenosine 5’-monophosphate; ND, not detected.

Supplementary Table S2

| Lipid Class | ^1^H Chemical shift | ^13^C Chemical shift |
| --- | --- | --- |
| Chol-C18 | 0.6931 | 12.05 |
| R-CH_3_ | 0.8231 | 19.86 |
| R-CH_3_ | 0.8386 | 20.72 |
| R-CH_3_ | 0.8677 | 22.82 |
| R-CH_3_ | 0.8857 | 14.18 |
| Chol-C21 (d) | 0.9261 | 18.89 |
| Chol-C9 | 0.9361 | 50.54 |
| Chol-C14 | 1.0031 | 57.14 |
| Chol-C19 | 1.0157 | 19.54 |
| Chol | 1.1022 | 56.24 |
| -CH=CH-CH_2_-**CH_3_** (t) | 0.9792 | 14.41 |
| Chol-C19 | 1.0155 | 19.53 |
| (CH_2_)_n_ | 1.2665 - 1.3116 | 22.89 - 32.16 |
| Chol | 1.5072 | 21.34 |
| F_β_:R-**CH_2_**-CH_2_-CO- | 1.609 | 25.13 |
| F_β_:CH=CH-CH_2_-**CH_2_**-CH_2_-CO- | 1.702 | 25 |
| Chol | 1.8115 | 31.48 |
| Chol | 1.8422 | 28.56 |
| Chol | 1.8506 | 37.61 |
| Chol | 1.9752 | 32.19 |
| -CH=CH-**CH_2_**- (18:1) | 2.0202 | 27.45 |
| Chol | 2.0233 | 40.08 |
| -CH=CH-**CH_2_**-CH=CH- (18:2;20:4) | 2.0554 | 27.38 |
| -CH=CH-CH_2_-CH_2_-**CH_2_**-CO- (20:4) | 2.1283 | 26.82 |
| Chol | 2.1743 | 36.78 |
| Chol | 2.2248 | 42.17 |
| Chol | 2.2543 | 42.21 |
| F_α_: R-CH_2_-**CH_2_**-CO- | 2.3256 | 34.48 |
| -CH=CH-**CH_2_**-**CH_2_**-CO- (22:6) | 2.4022 | 34.38 |
| -CH=CH-**CH_2_**-CH=CH- (18:2) | 2.7765 | 25.88 |
| -CH=CH-(**CH_2_**-CH=CH-)_y_ (20:4, 22:6) | 2.8142 | 25.87 |
| PE-2’ | 3.1025 | 41.00 |
| -N^+^(Me_3_)_3_ (PC) | 3.2163 | 54.45 |
| PI-5’ | 3.2319 | 74.92 |
| PS-1’ | 3.2842 | 39.54 |
| PI-3’ | 3.3932 | 71.71 |
| Chol-C3 | 3.4711 | 71.58 |
| PC-2’ | 3.5861 | 66.93 |
| PI-4’ | 3.6483 | 72.85 |
| PI-2’ | 3.6483 | 72.85 |
| Gly-C3 (DG) | 3.6998 | 60.97 |
| PI-6’ | 3.7848 | 72.21 |
| PI-1’ | 3.8821 | 77.1 |
| Cer-C2 | 3.9291 | 54.12 |
| Gly-C3(PLS/PC/PE) | 4.0034 | 64.01 |
| Cer-C1/Gly-C3(PI)/PE-1’ | 4.0361 | 61.61 |
| Cer-C3 | 4.1573 | 71.84 |
| Gly-C1 (DG/TG/PC/PE/PI) | 4.1665 | 62.96 |
| Gly-C3 (PI)/PE-1’ | 4.2426 | 59.24 |
| PC-1’ | 4.2431 | 59.21 |
| PLS-(=CH-) | 4.3584 | 108.26 |
| Gly-C1 (DG/TG/PC/PE/PI) | 4.4156 | 62.02 |
| RRC=CH_2_ | 4.6855 | 111.51 |
| Gly-C2 (DG) | 5.1694 | 72.09 |
| Gly-C2 (PC/PE/PI) | 5.2301 | 70.78 |
| Mono-UFA | 5.3492 | 130.18 |
| Poly-UFA | 5.3724 | 128.67 |
| PLS (-O-HC=) | 5.9158 | 145.07 |

**Table S2**. ^1^H NMR assignments performed in the lipid extracts of the cell lines. The chemical shifts are relative to the internal standard TMS (δ = 0.000 ppm). The bold assignments refer to specific resonance in the fatty acid chain. Chol, Cholesterol; F_β_,protons adjacent to the COOH group in the fatty acid chain; F_α_, protons adjacent to the COOH group in the fatty acid chain; PE, Glycerophosphoethanolamines; PC, Glycerophosphocholines; PI, Glycerophosphoinositols; PS, Glycerophosphoserines; DG, Diacylglycerol; Cer, Ceramide; TG, Triacylglycerol; PLS, Plasmalogen; UFA, Unsaturated fatty acid.
